# Galangin ameliorates PTU-induced vitiligo in zebrafish and B16F10 cells by increasing melanogenesis through activation of the p38/JNK MAPK pathway

**DOI:** 10.3389/fphar.2025.1521097

**Published:** 2025-03-10

**Authors:** Zulipikaer Wusiman, Ai-Mei Zhang, Shu-Shu Zhang, Ping-Ping Zhao, Yu-Tong Kang, Yun Zhang, Zhi-Jian Li, Shi-Xia Huo

**Affiliations:** ^1^ Uygur Medical Hospital of Xinjiang Uygur Autonomous Region, Urumqi, China; ^2^ Key Laboratory of Evidence-Based and Translation, Xinjiang Hospital preparation of Traditional Chinese Medicine, Urumqi, China; ^3^ The First Affiliated Hospital of Shihezi University, Shihezi, China; ^4^ Biology Institute, Qilu University of Technology (Shandong Academy of Sciences), Jinan, China

**Keywords:** galangin, zebrafish, B16F10 cells, tyrosinase, MAPK signaling pathway

## Abstract

**Objective:**

*Alpinia officinarum Hance* is a traditional herb in Xinjiang for the treatment of vitiligo, and galangin (GA) is a flavonoid isolated from its roots. However, its therapeutic mechanism remains unclear.

**Methods:**

In this study, 1-phenyl-2-thiourea (PTU) was used to establish a vitiligo model in zebrafish. After successful modeling, different concentrations of GA (1 and 2 μM) were administered, and the distribution of melanin granules was observed by assaying the melanin content, masson-fontana staining and tyrosinase activity. Transcriptomic analysis and molecular docking were used to identify potential GA-related pathways and targets for improving vitiligo. In addition, we evaluated the proliferation of B16F10 cells by PTU induction and also observed cellular melanin distribution using masson-fontana staining. Finally, Western blot was performed to detect the proteins of the relevant pathways.

**Results:**

The results showed that GA significantly increased melanin production and tyrosinase activity in depigmented zebrafish. In addition, we found that GA decreased ROS and MDA levels and increased the expression of GSH, CAT and T-SOD. In addition, transcriptome analysis indicated that GA likely acts through the mitogen-activated protein kinase (MAPK) signaling pathway. GA has a strong binding affinity for important targets.GA significantly increased the expression of genes such as mapk8b, mapk14a, mapk3, mitf, tyr, tyrp1b, tyrp1a, dct, and oca2, and decreased the expression of genes such as expression of genes such as raf1 and egfr. In addition, GA enhanced the viability of B16F10 cells, increased intracellular melanin content, and increased the expression of proteins such as p38, JNK1/2/3, TYR, MITF, TRP1, TRP2, and so on.

**Conclusion:**

GA increases melanin production and distribution, improves tyrosinase activity, upregulates the expression of related genes and proteins through activation of MAPK and tyrosine metabolic pathways, downregulates oxidative stress, and then regulates changes in melanin synthesis to improve vitiligo.

## 1 Introduction

Vitiligo is a skin disorder characterized by localized or generalized depigmentation of the skin, with a global prevalence of 0.5%–2% (Gopal et al., 2014). Depigmentation is a clinical sign of vitiligo. The clinical symptoms of this condition include the presence of white spots of varying sizes and shapes on the hands, neck, face, and other body parts. These spots may cause patients to experience significant appearance anxiety, leading to mental stress and a decline in their quality of life (Ezzedine et al., 2015; Li et al., 2017). Currently, the pathogenesis remains unknown, and it may be related to oxidative stress, melanocyte self-destruction, autoimmune, neuropsychiatric, and genetic factors (Namazi, 2007). The narrow-spectrum UVB, calcineurin inhibitors, local corticosteroids, and immunomodulators are commonly used in Clinical treatment means (Speeckaert and Van Geel, 2017; Karagaiah et al., 2020). However, these treatments can cause some side effects. Therefore, developing safe and effective drugs to treat vitiligo is highly significant.

Recently, studies have shown that the synergistic effect between oxidative stress and autoimmunity ultimately causes melanocyte damage or apoptosis and thus induces vitiligo (Xie et al., 2016). However, these theories are not sufficient to explain the pathogenesis of vitiligo alone. Normally, the body’s oxidative and antioxidant systems are in a relatively balanced state. external harmful stimuli caused by oxidation and antioxidant imbalance, resulting in oxidative stress, causing the local environment of the body to produce a large number of oxygen free radicals, making melanocyte damage or even apoptosis, thus leading to the occurrence of skin diseases (Colucci et al., 2015). Excessive production of reactive oxygen species (ROS) can lead to imbalance of the antioxidant system in melanocytes, ultimately causing cellular damage (Xuan et al., 2022). Although the cause of vitiligo is not clear, vitiligo causes melanocyte damage or death.

Melanin synthesis is affected by related enzyme-catalyzed and chemical reactions, among which Tyrosinase (TYR), as a key and limiting enzyme for melanin synthesis, has been considered as an important target for the study of therapeutic agents for melanin disorders. TYR catalyzes the hydroxylation of L-tyrosine to3,4-dihydroxyphenylalanine (L-DOPA) and the subsequent oxidation of DOPA to dopaquinone (Cordero and Casadevall, 2020). However, TYR is transcriptionally regulated by the microphthalmia-associated transcription factor (MITF), which is a major gene for early-stage melanoblast markers. In addition, MITF not only regulate TYR but also activate melanocyte differentiation and the expression of other related proteins, such as tyrosinase-associated protein 1 (TRP1) and tyrosinase-associated protein 2 (TRP2) (Ye et al., 2011). Therefore, following the expression of MITF, TYR, TRP1, and TRP2, the accelerated melanocyte differentiation resulted in increased melanin synthesis.

Zebrafish is a small but extremely powerful model organism, widely used for high-throughput drug screening and disease modeling (Yalcin et al., 2017). There is a high degree of similarity between zebrafish genes and human genes, 87% (Mushtaq et al., 2013). Similar to mammals, the pigment cells are derived from the neural crest in zebrafish at the embryonic stage. Zebrafish melanin is formed within 24 h after fertilization. Zebrafish have the advantages of fast development, transparent embryos, and easy observation of body surface pigment, which is widely used in cosmetic whitening efficacy evaluation (Cooper, 2017). It is well known that 1-phenyl-2-thiourea (PTU) is an inhibitor of tyrosinase and is widely used as a chemical agent to inhibit zebrafish hyperpigmentation and melanin secretion by melanocytes (Higashi et al., 2000; Eom et al., 2022). In this study, PTU was used to construct a depigmentation model.

Galangin (GA) is a flavonoid compound isolated from the dried rhizome of *Alpinia officinarum Hance*, which has anti-inflammatory, antioxidant, antibacterial, anti-tumor, and skin protection activities (Lee et al., 2017; 2022; Abukhalil et al., 2021; Hassanein et al., 2023). In a previous study, GA was found to upregulate TYR protein expression in C57BL/6 mouse tissues and increase the number of basal melanocytes and melanin-containing epidermal cells in mouse skin (Huo et al., 2014). However, the role of GA in regulating melanin synthesis remains unclear. In this study, we utilized PTU along with zebrafish and B16F10 cells to develop a depigmentation model. With the help of this model, we examined the impact of GA on melanin synthesis.

## 2 Materials and methods

### 2.1 Reagents and antibodies

Galangin (CHB180610, Chengdu, China) was purchased from Chengdu Crooma Biotechnology Co., Ltd., 8-methoxypsoralen (8-MOP, C12393442, Shanghai, China) was purchased from Shanghai Maclin Biochemical Technology Co., Ltd., PTU (103-85-5, Sigma). All antibodies used in this study are listed below: β-actin (AF7018), TYR (AF5491, Affinity), MITF (AF6027, Affinity), TRP1 (DF13325, Affinity), TRP2/dct (AF5303, Affinity), P38 (AF6456, Affinity), JNK1/2/3 (AF6318, Affinity), ERK1/2 (AF0155, Affinity), p-p38 (AF4001, Affinity), p-JNK1/2/3 (AF3320, Affinity), p-ERK1/2 (AF1015, Affinity). The secondary antibodies, namely anti-rabbit (S0001, Affinity).

### 2.2 Animals and maintenance

According to standard procedures, Wild-type AB zebrafish were maintained and housed at 28°C under 14 h light/10 h dark conditions. The day before mating, male and female zebrafish were placed in spawning tanks in a 2:2 ratio. Zebrafish embryos were obtained from 10:00–11:00 a.m. on the day of mating and washed 3 times with water. Zebrafish embryos were transferred to zebrafish medium containing 0.0001% methylene blue (0.4 mmol/L CaCl_2_, 5 mmol/L NaCl, 0.17 mmol/L KCl, 0.16 mmol/L MgSO_4_) and incubated at 28°C under light control. All experiments were carried out in compliance with the standard ethical guidelines and under the control of the Biology Institute, Qilu University of Technology Animal Ethics Committee.

### 2.3 Generation of depigmentation model and administration of GA

Selected wild-type AB zebrafish 24 h after fertilization were treated with different concentrations (50 and 200 μM) of PTU for 24 h. The zebrafish were observed for death, malformation, and melanin changes under the somatic microscope. According to the safety pre-evaluation of GA and reference (Karunarathne et al., 2019) of Method, the blank control group was treated with fish medium, and the remaining groups were treated with 200 μM PTU solution for 24 h. The blank control and model groups were added to the fish medium, the positive drug group (25 μM 8-MOP), and different concentrations (1, 2 μM) of GA were treated for 48 h.

### 2.4 *In Vivo* tyrosinase activity and melanin content assay

Tyrosinase is a key enzyme in the synthesis of melanin. For the measurement of tyrosinase activity, zebrafish larvae from the above interventions cultured in EP tubes were sonicated in lysis buffer at 4°C, and then precipitation was performed with Sorvall™ ST16 centrifuges (Thermo Fisher Scientific, United States) using 11,000 rpm at 4°C for 10 min. Protein concentrations were determined by the BCA kit with bovine serum albumin (P0010, Beyotime) as a standard and operated according to the instructions of the tyrosinase kit (BC4055, Solarbio^®^). The optical density was measured at 475 nm.

Melanin granule was dissolved in 1M NaOH (with 10% DMSO) for 2 h at 80°C, and solubilized melanin was measured at 405 nm. Then, the relative content of melanin was exhibited as a percentage of the control.

### 2.5 Fontana-Masson staining

We densitometrically analyzed melanin in zebrafish and used Fontana-Masson staining (Kim et al., 2018). Paraffin sections were placed on slides and stained with Fontana silver-ammonia solution for 1 h at 56°C, then rinsed. Then, the solution was added to prepare the sections. Sections are fixed in the cryogenic solution for 5 min and then rinsed. Then, neutral solution is added for 5 min and rinsed 3 times. Finally, after gradient dehydration with ethanol, the sections were eluted with xylene and fixed with neutral gel.

### 2.6 *In Situ* hybridization (ISH) assay

According to the experimental method of reference (Liu et al., 2020; Lai et al., 2021). After drug incubation and prehybridization, 5% BSA solution (SW3015, Solaebio) was closed at room temperature for 2 h, and Digoxin antibody (32871922) was added and incubated at 4°C overnight. Then, each group was washed twice with alkaline AP buffer, and 500 μL BM purple AP (62321800) dyeing solution was incubated for 30 min in a dark environment; the dyeing solution was sucked out, cleaned with PBST solution 5 times, and fixed with 4% paraformaldehyde for 20 min. Zebrafish were immersed in 100% glycerol solution (GB20000), placed under a stereomicroscope, and photographed.

### 2.7 ROS detection

After the GA intervention, zebrafish were washed three times with PBS buffer, and each experimental group was incubated with zebrafish culture water containing 10 μM DCFH-DA probe at room temperature and protected from light for 30 min. At the end of the incubation, zebrafish were washed three times with zebrafish culture water, anesthetized by adding 0.3% tricaine, and then suctioned onto slides containing 4% methylcellulose solution, and zebrafish were fixed in a bilateral position. The zebrafish were fixed on the slide in a lateral position with both eyes facing each other, and placed under a fluorescence microscope to take pictures and count the fluorescence intensity of the zebrafish body surface.

### 2.8 Evaluation of the level of oxidative stress

The oxidation indicators malondialdehyde (MDA, BC0025), catalase (CAT, A007-1-1), glutathione (GSH, BC1175), and total superoxide dismutase (T-SOD, A001-1) levels in zebrafish organization were determined using the ELISA kits. All tests were carried out following the kit instructions.

### 2.9 Transcriptome analysis

#### 2.9.1 Transcriptome sequencing sample collection and preparation

After the medication was finished, the blank control group, model group, and galanin group were washed out twice using PBS and transferred into a 1.5 mL centrifuge tube. 60 zebrafish were collected in each group. The whole RNA extraction, transcriptome sequencing, and analysis process was commissioned by Qingdao Ouyi Biotechnology Co., LTD.

#### 2.9.2 Differential expression gene screening and enrichment analysis

The DESeq2 software was used to analyze the difference expression between groups, calculate the difference multiples and difference significance, and test the difference significance by using the negative binomial distribution method. Differentially expressed genes were screened according to |log_2_FC| >1.5 and *p* < 0.05, and the intersection of differentially expressed genes was identified as co-expressed genes. Then, cluster analysis, GO enrichment analysis, and KEGG function analysis of the differential transcripts were performed to determine the biological functions or pathways affected by the differential transcripts.

### 2.10 Molecular docking

To elucidate the binding activity of GA to its potential targets against depigmentation, the binding ability of GA to the Key genes in the MAPK signaling pathway in the transcriptome sequencing was verified by molecular docking. The mol2 format file of GA was downloaded from the TCMSP database, and the crystal structure of the core target protein receptor was downloaded from the RCSB PDB database. Ligand small molecule and core protein structures were pre-processed using Discovery Studio 2016 software, followed by molecular docking. The docking results are given by binding energy (kJ/mol). The smaller the binding energy, the higher the binding activity of the predicted component to the target.

### 2.11 Real-time quantitative PCR anaylsis

After treatment, each group zebrafish were collected to extract RNA from each group using the SPARKeasy Tissue/Cellular RNA Rapid Extraction Kit. Then 1 μg RNA samples were taken from each group for reverse transcription according to HiScript^®^ ⅡQ RT Super Mix for qPCR (+gDNA wiper) kit. Finally, the ChamQ Universal SYBR qPCR Master Mix kit was used for qPCR detection, with β-actin as the internal reference gene. The primer sequences were in Table 1.

**TABLE 1:** Primers sequence.

Gene	Nucleotide sequence
*mapk14a*	Forward:GCCATGAGGCTCGTACTTACAT
	Reverse:AGCCTCTGCTGCTGTAATCC
*mapk8b*	Forward:TGCTGGCATCATACACAGGG
	Reverse:GGCCCGATAATAGCGTGTCA
*akt2*	Forward:AAAGTCCCGCACCAAAGTGA
	Reverse:GCGTAGGATCTTCATGGCGT
*mapk3*	Forward:CACAGCAACTCAGCAACGAC
	Reverse:TCCTCGCCAACCCAAAATCA
*akt1*	Forward:AAGAGGGGATCACAGACGGA
	Reverse:CATCACCACCCCTAAACCCC
*egfr*	Forward:GACGACCGCATGCATTTACC
	Reverse:TTCAGGCTCACAGAGTGCAG
*raf1*	Forward:CATGTCTCCAATTACACCAGCC
	Reverse:ACACCAGGAGCTTGGAGATG
*gsk3β*	Forward:CCATCCATGGACTAAGGTGTTTC
	Reverse:ACATTTGGTTCCCGCAGTTC
*tyr*	Forward:TCCTCTGTGTTCTCATCCT
	Reverse:TGAAGTATCCGTCGTTGTG
*tyrp1a*	Forward:CTCATCATCGTCGCCATC
	Reverse:GAACCTCCTGAAGAACACA
*tyrp1b*	Forward:CAGTGGTGCTGGTTGTAG
	Reverse:TGGCTGTATTCTCAATGTCT
*mitf*	Forward:GGACAACCACAACCTCAT
	Reverse:CCACTAACTCAGCGGAATA
*dct*	Forward:CTGTGACCAATGAGGAGATT
	Reverse:CATAGGATTTGGGACTGTGT
*oca2*	Forward:CAGTCAAGATGCCGATGT
	Reverse:TCCAGTGAAGTCCGATGT
*silv*	Forward:GCAGAAGACACAGTTATCG
	Reverse:CAGCATCACCACATTATTCA
*β-actin*	Forward:CCTAGAAGCATTTGCGGTGG
	Reverse:AAGAGCACAAGAGGAAGAGAGAGAC

### 2.12 Cell culture and cell viability assay

B16F10 cell (FH0361) was Purchased from Shanghai Fuheng Biotechnology Co., LTD. The cells were cultured in a sterile and 37°C, 5% CO_2_ culture environment. B16F10 cell was incubated in RPMI1640 medium (2301522, Bioexplorer) supplemented with 10% FBS (2205117, Bioexplorer) and 1% penicillin/streptomycin solution (2112871, Bioexplorer). Then, the culture was maintained at 37°C in a constant temperature incubator with 5% CO_2_.

For the cell viability, the CCK-8 assay (30327010104, Genview^®^) was performed. The experimental groups were such as the blank control group (medium), model group (200 μM, PTU), positive drug group (PTU+25 μM, 8-MOP), and GA treatment group with different concentrations (PTU+1, 2 μM GA) incubated for 24 h. Then CCK-8 is added to the wells and incubated at 37°C for 1.5 h. The cell viability is measured using a spectrophotometer at 450 nm. Cell viability was measured using a spectrophotometer at 450 nm absorbance (BIO-GENE, Guangzhou, China).

### 2.13 *In Vitro* Fontana-Masson staining and melanin content assay

After treatment, same procedure as “2.5.” Placed under SZX16 Microscope (Olympus, Tokyo, Japan) for photography and analysis of melanin staining.

B16F10 cells were collected and added to incubate cell lysate for 30 min at 4°C. Then, each group was added 1 mol/L NaOH/10% DMSO for 2 h at 80°C, and the solubilized melanin was measured at 405 nm.

### 2.14 Western blotting analysis

After treatment, washing each group was performed two times with PBS solution, followed by lysis of the cells with RIPA/PMSF lysis buffer (110,414). The supernatant was collected, and its protein concentration was measured using BCA protein assay reagents. 5 × SDS-PAGE loading buffer was added to each group of proteins and denatured at 95°C for 10 min. Then, the equal amount of protein was subjected to 12% SDS–PAGE gel electrophoresis and transferred onto PVDF membranes. After blocking with 5% skim milk in TBST for 2 h at room temperature, membranes were incubated overnight at 4°C with primary antibodies against. And the second day, the membrane was washed 3 times with 1 × TBST and incubated with anti-rabbit IgG (H + L) HRP (S0001) at room temperature for 2 h. Finally, the immunoblotting chemiluminescent reagent (KF8005, Affinity) was added to develop the image. The grayscale value of proteins was calculated and analyzed using ImageJ software.

### 2.15 Statistical analysis

The results were all analyzed using One-way ANOVA and presented as mean ± SEM with a significance level of *p <* 0.05. Each experiment was done at least three times. #*p <* 0.05, ##*p <* 0.01, ###*p <* 0.001 and ####*p <* 0.0001 vs. the control group, *p < 0. 05, **p < 0.01 and ***p < 0.001 vs. the model group.

## 3 Results

### 3.1 PTU-induced zebrafish depigmentation model

**FIGURE 1:**
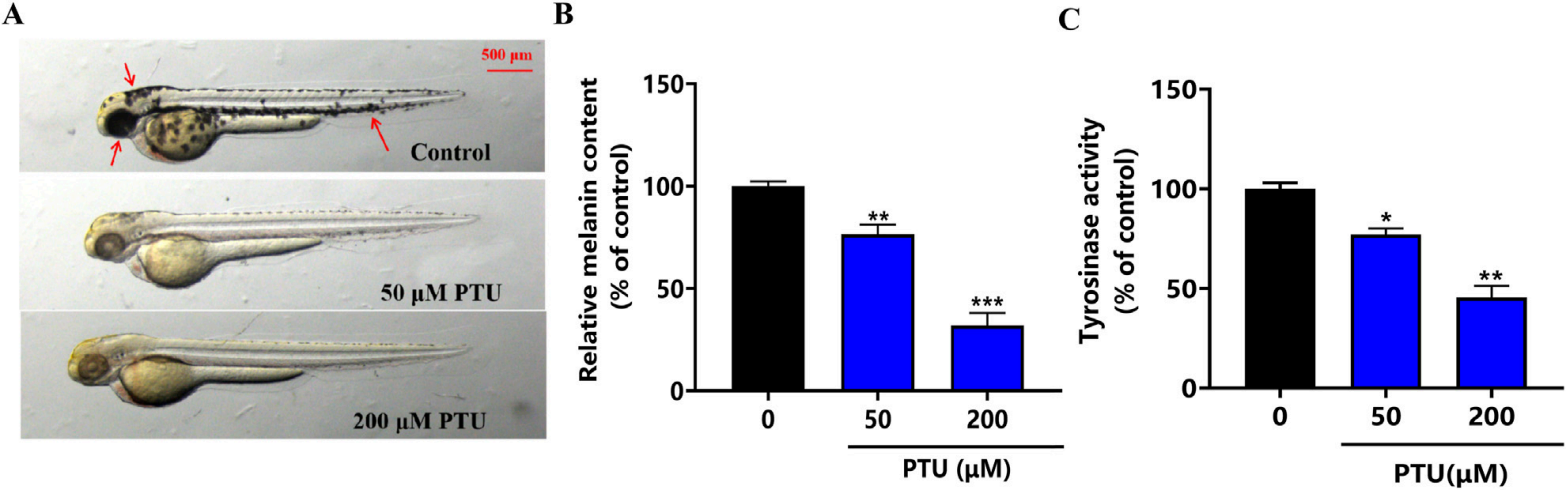
PTU-induced zebrafish depigmentation model. **(A)** the phenotype of the depigmentation model constructed by PTU (n = 10, size: 500 μM). **(B)** Histogram of melanin content, and **(C)** the histogram of tyrosinase activity (n = 10).

The results showed that a PTU of 50 and 200 μM inhibited zebrafish body pigmentation, which was similar to the symptoms of patients with depigmentation at different stages of clinical practice. After that, melanin content and tyrosinase activity were significantly inhibited at 200 μM (Figures 1A–C). Therefore, 200 μM PTU was used as the dose to construct the depigmentation model.

### 3.2 Effect of GA on melanin synthesis and TYR activity of zebrafish

**FIGURE 2:**
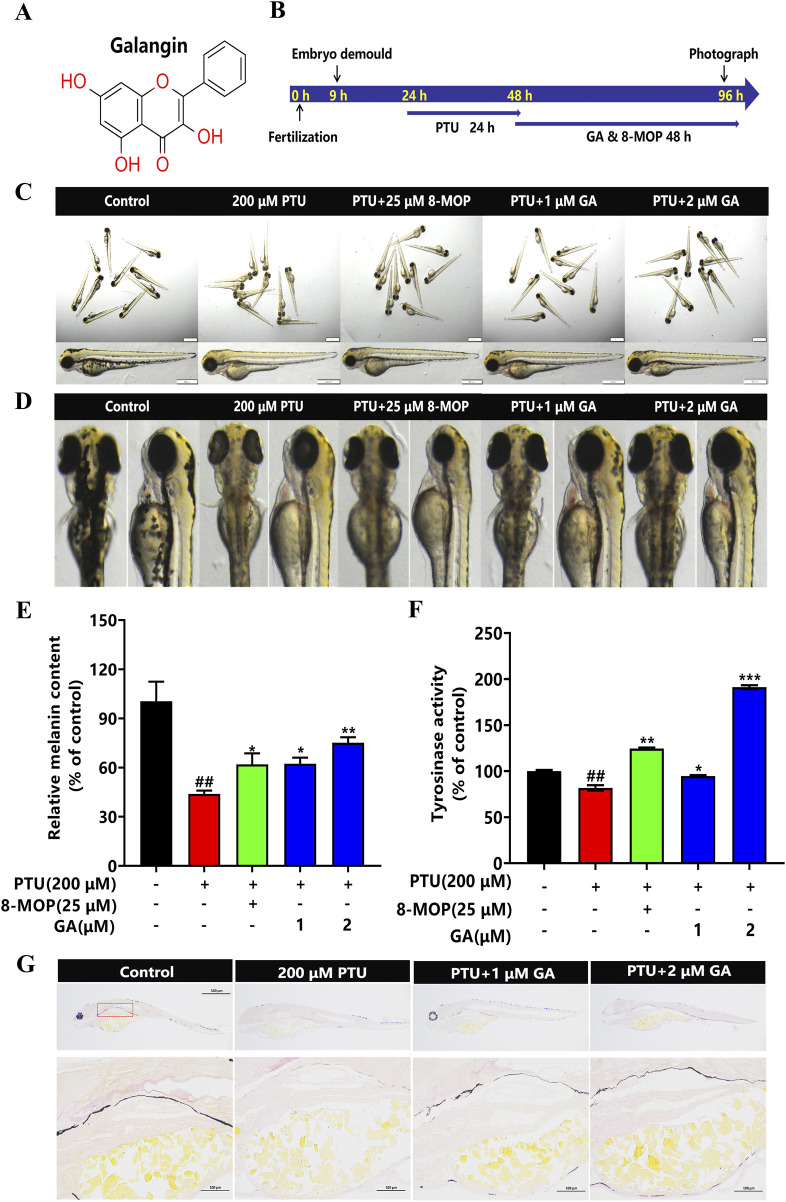
Effect of GA on melanin synthesis and TYR activity of zebrafish. **(A)** Chemical structure of GA. **(B)** The nine hpf zebrafish embryos were demembrane with strepsin E, then 200 μM PTU was added to the zebrafish embryos at 24 hpf for 24 h, and GA at different concentrations (1 and 2 μM) was added to the zebrafish embryos for 48 h. **(C)** The overall morphology of Zebrafish (n = 10, size:1 mm, 500 μm). **(D)** Morphological plots of the back and side of the zebrafish. **(E)** Relative melanin content and Relative tyrosinase activity **(F)** were calculated by normalizing with the control group (n = 10). **(G)** Fontana-masson staining (n = 10, size: 100 μm).

To investigate the effects of GA (Figure 2A) on melanin synthesis in zebrafish, we used 200 μM PTU solution to reduce pigmentation in zebrafish (Figure 2B). The intervention of different concentrations (1 and 2 μM) of GA had no effect on morphology, mortality, and malformations in zebrafish (Figures 2C, D). The results of the assay of melanin content and tyrosinase activity in zebrafish showed that GA could increase melanin synthesis and tyrosinase activity in zebrafish (Figures 2E, F). furthermore, we used masson-fontana ammonia-silver staining to evaluate pigmentation areas, the results showed that the melanin particles in the GA group were significantly higher than those in the model group in a dose-dependent manner, such as red dotted areas (Figure 2G). It is suggested that GA can significantly increase the melanin content and TYR activity of zebrafish.

### 3.3 Effects of GA on content of ROS and oxidation indexes

**FIGURE 3:**
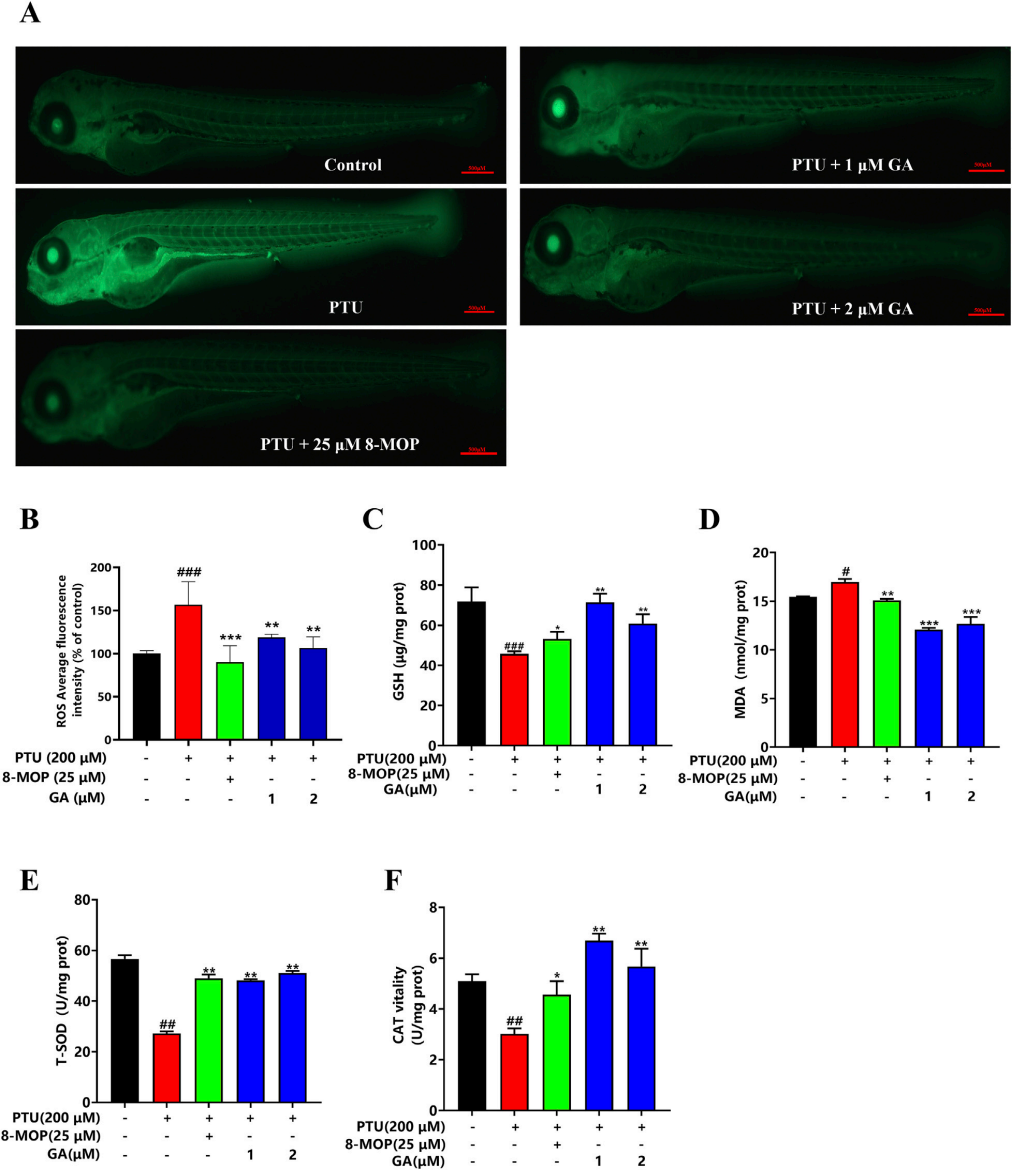
Effects of GA on content of ROS and oxidation indexes in zebrafish. **(A)** Zebrafish ROS fluorogram (n = 10, size: 500 μm). **(B)** ROS content in zebrafish. **(C–F)** GSH, MDA, T-SOD, CAT content measurement (n = 10).

The experimental results showed that the ROS content in zebrafish from the PTU model group was significantly increased compared with that in the blank control group, and the ROS content in zebrafish from the GA intervention groups was significantly lower than that in the PTU model group in a dose-dependent manner, which was in line with the results of the 8-MOP-positive drug. This suggests that GA can reduce the accumulation of ROS produced by PTU, inhibit the oxidative stress damage to melanocytes, and thus play a role in promoting melanin synthesis (Figures 3A, B).

Melanin is synthesized by tyrosinase through a series of oxidation and reduction reactions, redox imbalance will cause the degradation of tyrosinase and lead to the reduction of melanin synthesis. To verify whether GA could protect melanin synthesis through antioxidant effect, MDA, CAT, GSH and T-SOD were verified. It was found that GA could significantly decrease the content of MDA and significantly increase the content of CAT, GSH and T-SOD (Figures 3C–F).

### 3.4 Transcriptome sequencing

**FIGURE 4:**
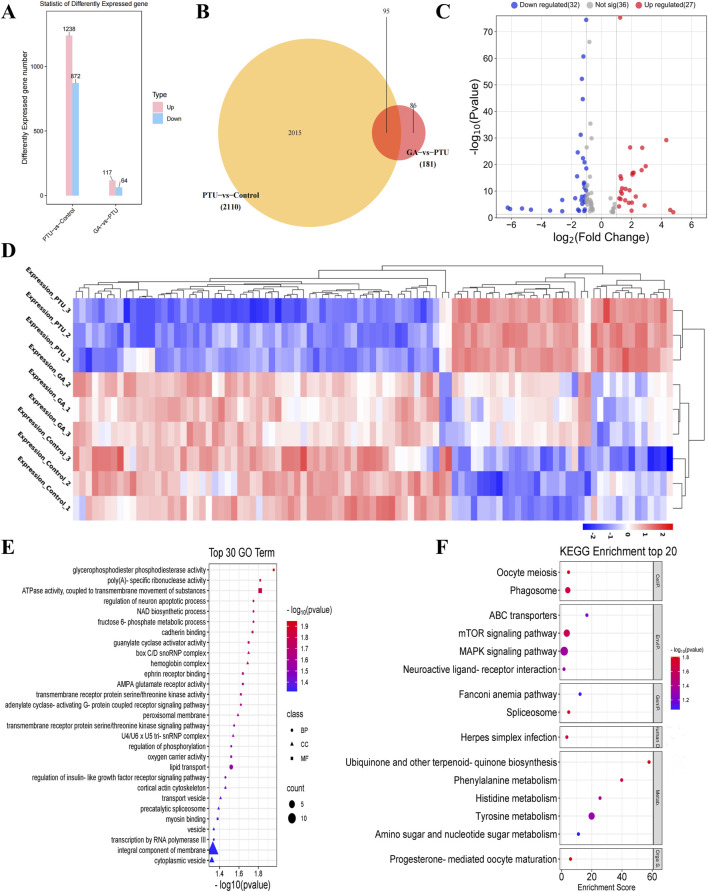
Analysis of differentially expressed genes (n = 3). **(A)** histogram of differentially expressed genes. **(B)** Venn diagram of differentially expressed genes. **(C)** Volcano plots of differentially expressed genes. **(D)** Cluster analysis of differentially expressed genes. **(E)** Gene Ontology (GO) functional enrichment analysis, biological processes (BP), cellular components (CC) and molecular functions (MF). **(F)** KEGG pathway enrichment analysis.

In the transcriptome analysis, as compared to the control, 2110 differentially expressed genes (DEGs) were detected in PTU-treated larval fish, including 1238 upregulated and 872 downregulated ones (Figure 4A). Alternatively, compared to PTU-induced larval fish, 181 (117 upregulated and 64 downregulated ones) were observed at concentrations of 1 μM GA (Figure 4B). After that, the 2110 and 181 differentially expressed genes screened were subjected to Venn diagram analysis, and 95 differentially expressed genes were obtained (Figure 4C). Finally, cluster analysis was performed on 95 differentially expressed genes (Figure 4D), and it was found that the GA group was similar to the Control group in terms of differentially expressed genes.

95 DEGs were then subjected to the Gene Ontology (GO) functional enrichment analysis, with |log2FC| > 1.5 as the screening conditions. Biological processes include metabolic processes, NAD biosynthetic processes, transmembrane receptor protein serine/threonine kinase signaling pathways, Cellular components include peroxisome membrane, U4/U6 x U5 trissnRNP complex, membrane components, cytoplasmic vesicles, Molecular functions include ATPase activity, guanylate cyclase activator activity, AMPA glutamate receptor activity, transmembrane receptor protein serine/threonine kinase activity (Figure 4E).

Thereafter, we performed pathway analysis with the coding genes of differentially expressed proteins using KEGG (Kyoto Encyclopedia of Genes and Genomes) database |log2FC| > 1.5 as the screening condition, including MAPK signaling pathway, mTOR signaling pathway, Tyrosine metabolism and phagosome, etc (Figure 4F).

### 3.5 Molecular docking

**FIGURE 5:**
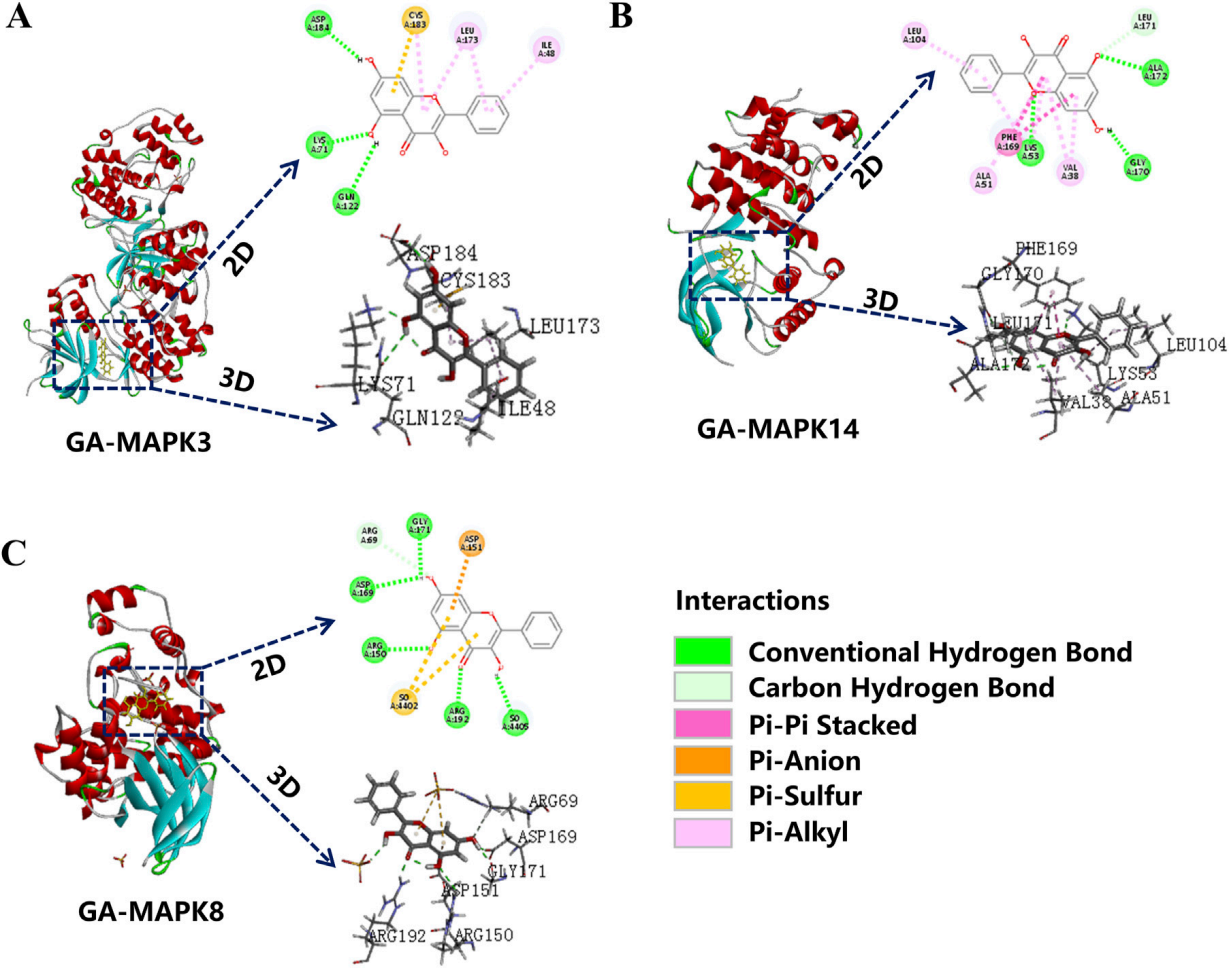
Molecular docking. **(A)** MAPK8; **(B)** MAPK14; **(C)** MAPK3 molecular docking with GA.

Transcriptome analysis results were enriched for the MAPK signaling pathway. Therefore, it was investigated whether GA exerts its anti-depigmentation effects by regulating key genes of the MAPK signaling pathway, molecular docking of its key genes showed that GA was well bound to MAPK14, MAPK8, and MAPK3, among which the lowest binding energy of MAPK14 represented a certain binding force (Table 2), indicating that MAPK signaling pathway is crucial in GA treatment of depigmentation disease (Figures 5A–C).

**TABLE 2:** Parameters analyzed by molecular docking.

Receptor protein	PDB ID	(KJ/mol) (-CDOCKER_energy)
MAPK3	6GES	−31.6697
MAPK14	5WJJ	−36.2279
MAPK8	3PZE	−24.927

### 3.6 GA reversed depigmentation through MAPK signaling pathway

**FIGURE 6:**
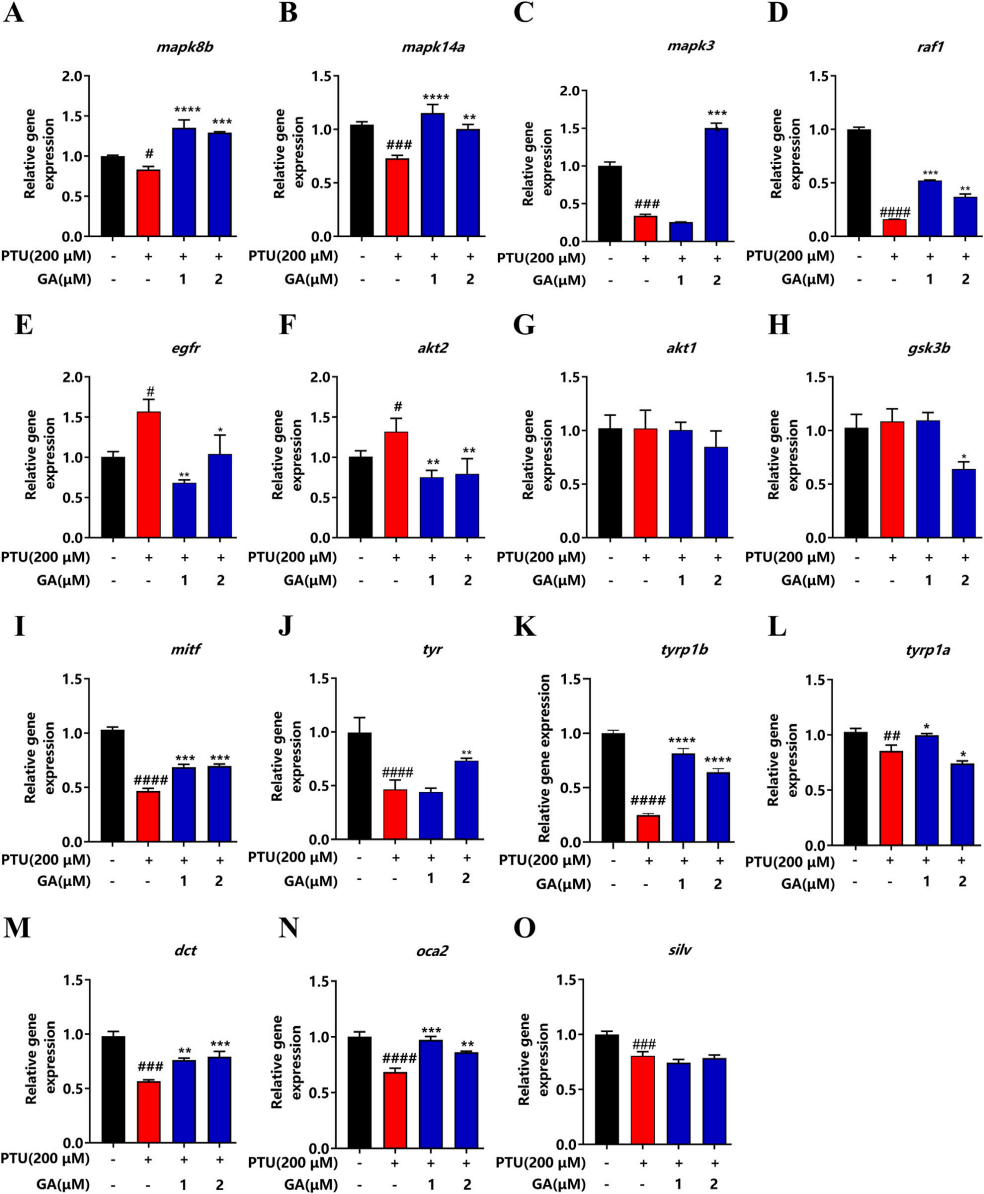
Effect of GA on expression levels of depigmentation-related genes in zebrafish (n = 10). **(A)** mapk8b; **(B)** mapk14a; **(C)** mapk3; **(D)** raf1; **(E)** egfr; **(F)** akt2; **(G)** akt1; **(H)** gsk3b; **(I)** mitf; **(J)** tyr; **(K)** tyrp1b; **(L)** tyrp1a; **(M)** dct; **(N)** oca2; **(O)** silv.

To verify the involvement of the MAPK signaling pathway and Common melanin genes in KEGG pathway enrichment analysis, we performed qPCR to compare the gene expression level of qualification of key factors. Compared with the model, the genes *mapk8b*, *mapk14a*, *mapk3*, *raf1*, *mitf*, *tyr*, *tyr1b*, *dct*, and *oca2* were significantly elevated and the genes *egfr*, and *dct2* were decreased after GA administration (Figures 6A–O).

### 3.7 Effect of GA on PTU-induced melanin synthesis in B16F10 cells

**FIGURE 7:**
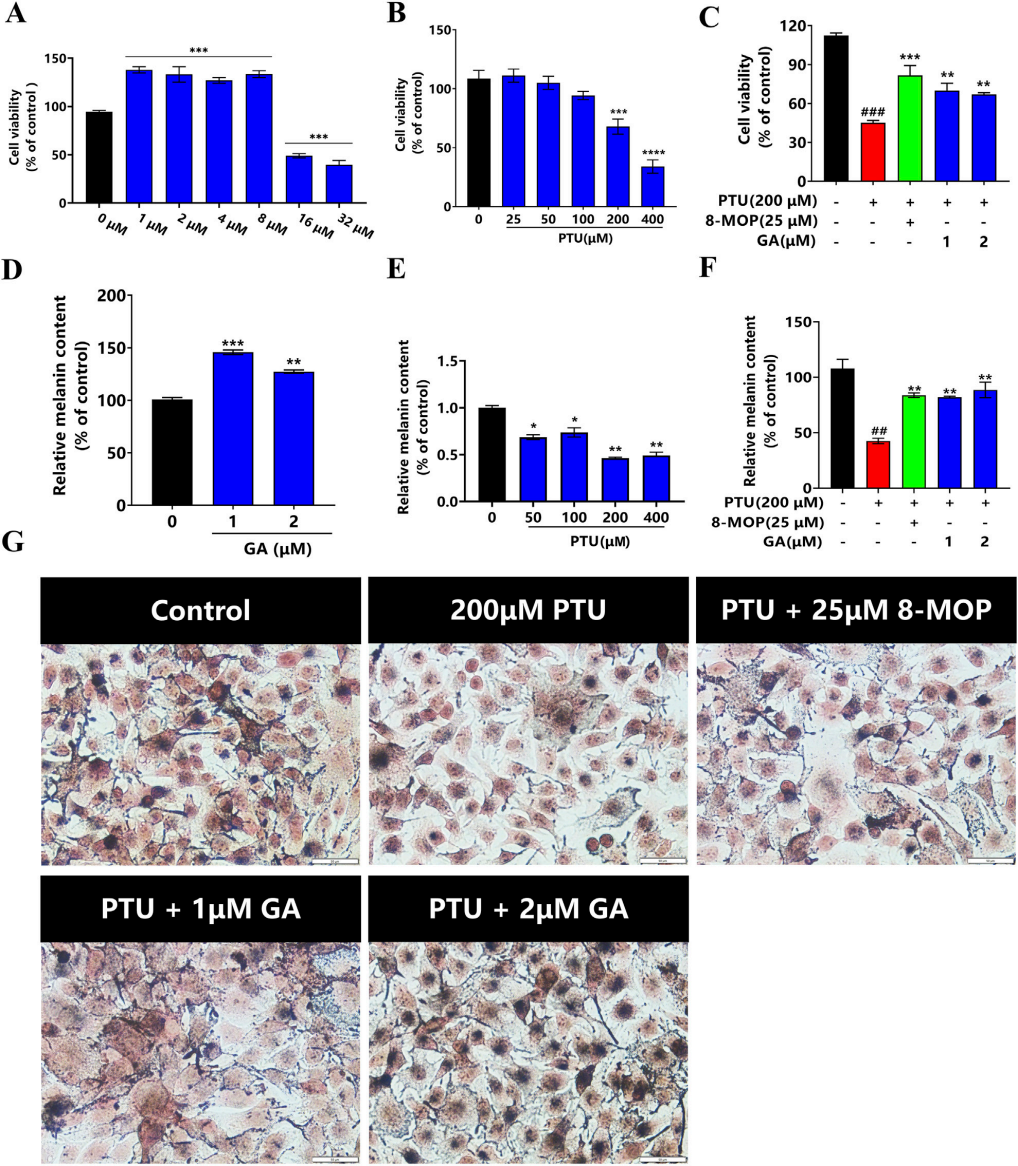
Protective effect of GA on B16F10 cells *in vitro* (n = 6). **(A)** B16F10 cells were incubated with GA at various concentrations for 24 h. **(B)** B16F10 cells were incubated with PTU at various concentrations for 24 h, and the cell viability was examined by CCK-8 assay. **(C)** Protective effect of GA on PTU-induced B16F10 cell damage. **(D)** After B16F10 cells were treated with different concentrations of GA and PTU for 24 h, the content of melanin was detected as GA and PTU **(E)**. **(F)** Protective effect of GA on PTU-induced melanin synthesis. **(G)** Fontana-masson staining of B16F10 cells (size:50 μM).

For the purpose of investigating the cyclotoxicity of GA *in vitro*, B16F10 cells were treated with different doses of GA (0, 1, 2, 4, 8, 16, and 32 µM) for 24 h. The results of CCK-8 indicated that GA was not cytotoxic to B16F10 cells at 24 h (1–8 µM), and 16 μM GA was cytotoxic (Figure 7A). Next, we determined whether PTU induced a decrease in B16F10 cell proliferation. After exposing the cells to various doses of PTU (25, 50, 100, 200, and 400 µM) for 24 h, the results showed that a dose-dependent decrease in cell viability was observed after PTU exposure (Figure 7B). Then, we investigated the protective effect of GA on PTU-exposed cells. The results revealed that GA was able to protect B16F10 cells from PTU-induced cell death, and therefore the doses of 1 and 2 µM were opted for subsequent experiments (Figure 7C).

We conducted a quantitative analysis of the melanin synthesis of GA and PTU, respectively. the results showed that GA (1 and 2 μM) significantly increased cellular melanin synthesis (Figure 7D). Melanin content diminished in a dose-dependent way after exposure to PTU (Figure 7E). Then, we examined the melanin-protective effect of GA on cells exposed to PTU. The results showed that GA protected B16F10 cells from PTU-induced melanin reduction (Figures 7F, G).

### 3.8 GA increases the expression of MITF and TYR by activating the MAPK signal pathway in B16F10 cells

**FIGURE 8:**
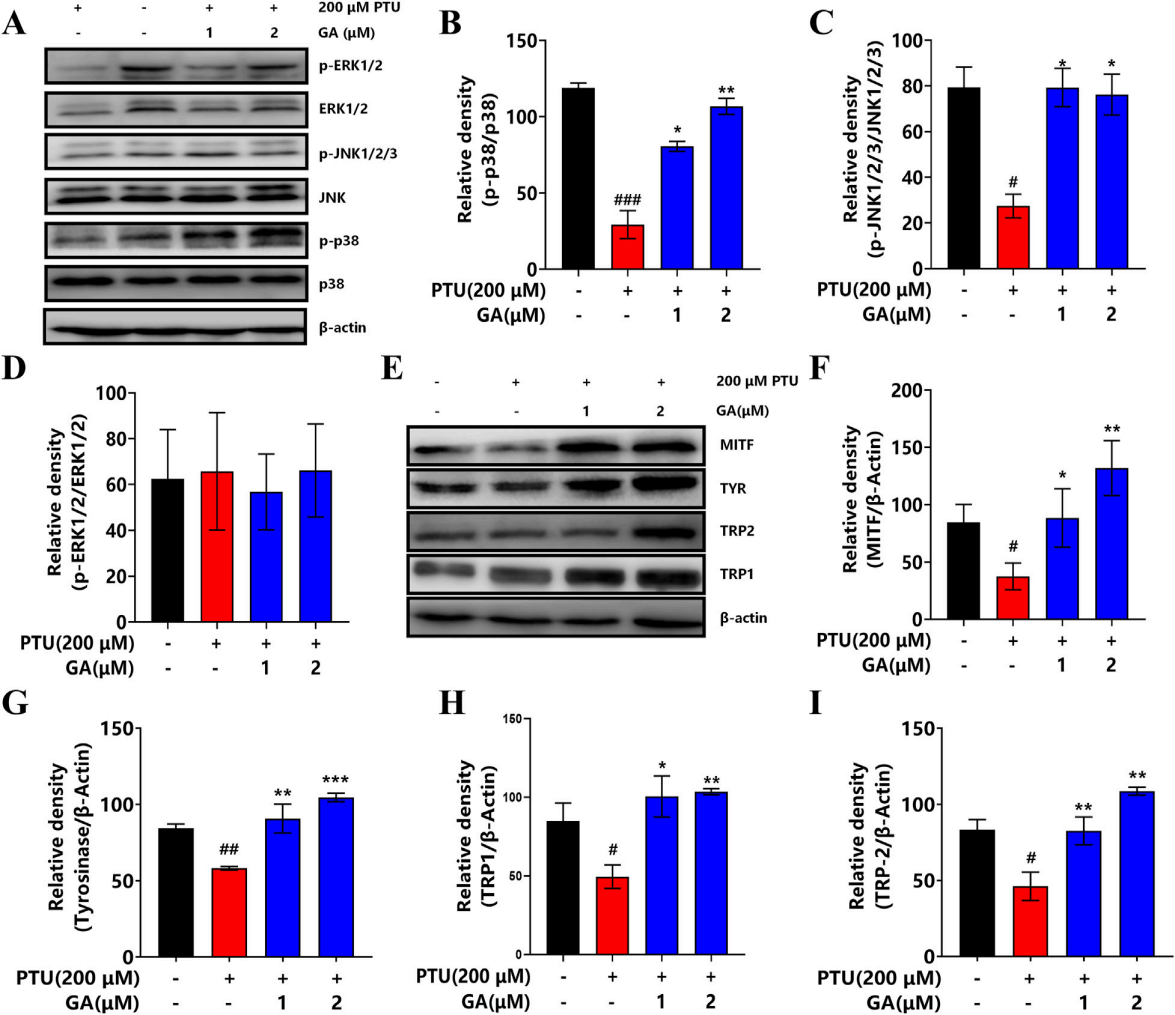
Effect of GA on the expression of key signaling pathway proteins. **(A)** Western blotting to detect the expression levels of p38 MAPK, ERK and JNK. **(B–D)** Effect of GA on the expression of p38, JNK1/2/3 and ERK1/2 proteins in B16F10 cells (n = 3). **(E)** Western blotting to detect the expression levels of MITF, TYR, TRP2 and TRP1. **(F–I)** Effect of GA on MITF, TYR, TRP2 and TRP1 protein expression in B16F10 cells (n = 3).

In order to further elucidate the effect of GA on the mechanism of melanin synthesis, the MAPK intracellular signal transduction cascade and TYR, MITF, TRP1, TRP2 were detected by Western blotting. The results showed that after GA intervention, the phosphorylation levels of p38 and JNK1/2/3 protein increased significantly, while ERK1/2 had no effect (Figures 8A–D). In addition, TYR, MITF, TRP1, and TRP2 protein expression was significantly higher than that in the model group after the GA intervention (Figures 8E–I). These results indicate that GA enhances melanin synthesis and is regulated by the phosphorylation expression of P38 MAPK and JNK, thereby activating melanocyte differentiation and melanin synthesis.

## 4 Discussion

Vitiligo is a kind of skin disease that is easy to diagnose, difficult to treat, and easy to relapse. It can occur at any age, and its clinical manifestations are mainly depigmentation. However, melanin is a biological pigment that is the main component of the color of human eyes, hair, and skin (Bonaventure et al., 2013), which can effectively absorb ultraviolet rays, reduce the damage of ultraviolet rays on human skin, and remove free radicals (Brenner and Hearing, 2008; Solano, 2020). Thus melanin plays a dual protective role in skin homeostasis. Studies have shown that PTU is a melanin inhibitor, which inhibits the conversion of tyrosine to melanin by reducing the activity of tyrosine hydroxylase, thus blocking the synthesis of melanin, and is commonly used to prepare the model of depigmentation (Li et al., 2012). In this study, two models of zebrafish and B16F10 cells were used to investigate the anti-depigmentation effect of GA and its mechanism.

We used the NaOH cleavage method and Fontana-Masson staining to detect the effects. The results showed that after the PTU modeling, the melanin content in the model group was significantly reduced compared to the blank control group. This indicates that the PTU dose of 200 μM can successfully induce the establishment of a depigmentation model. However, after GA intervention, the melanin content was significantly higher than that in the model group. Therefore, the study confirms that GA can promote pigmentation in zebrafish and may be a potential drug for the treatment of depigmentation.

Oxidative stress has been widely regarded as a key factor in initiating melanocyte damage, and excessive oxidative stress can cause free radical metabolism disorder and ROS accumulation in the body, resulting in melanocyte damage (He et al., 2022; Xuan et al., 2022). However, melanin is formed by converting L-tyrosine into L-3,4-dihydroxyphenylalanine (L-DOPA) by tyrosinase, and converting L-DOPA to dopaquinone, which undergoes a series of oxidation and reduction reactions (Cordero and Casadevall, 2020). In addition, T-SOD, CAT, and GSH are important antioxidant enzymes in the body, which play an important role in the balance of oxidation and antioxidants in the body, scavenging free radicals and protecting melanocytes from damage (Passi et al., 1998). The decrease of T-SOD, CAT, and GSH content will result in a decrease in antioxidant capacity, which leads to oxidative damage of melanocytes (Mathachan et al., 2021). MDA is one of the products of lipid peroxidation, and the level of MDA can reflect the damage degree of lipid peroxidation. The higher the level of MDA, the faster the oxidative damage of cells (Karsli et al., 2014). In our study, the levels of antioxidant enzymes and oxidative enzymes were measured by ELISA. The findings revealed that the activity of tyrosinase, T-SOD, CAT, and GSH contents significantly increased, while MDA contents significantly decreased after administering GA. These results suggest that GA may aid in the restoration of zebrafish pigmentation by enhancing the body’s ability to resist oxidative stress.

Transcriptome sequencing can study gene function and gene structure from the whole level and reveal specific biological processes (De Jong et al., 2019; Kleino et al., 2022). In the KEGG pathway analysis result, we observed a significant change in the MAPK signaling pathway. According to literature reports, the MAPK signaling pathway plays an important role in melanin synthesis (Zang et al., 2022). Zhou et al. (2012) found that alcohol extract from Vernonia anthelmintica (L.) promoted the melanin synthesis of B16F10 cells and primary melanocytes by activating the p38 MAPK signaling pathway. Studies suggest that MR-21 suppresses the effects of melanin synthesis by activating EGFR expression (Lin et al., 2017). 23R-AMA downregulates the expression of mitf in B16F10 cells by inhibiting the c-Raf/ERK signaling axis (Kaneda et al., 2019). Studies have found that PA and EP activate the PI3K/AKT signaling pathway by inducing GSK3β phosphorylation, resulting in reduced melanin synthesis (Zhou and Sakamoto, 2019). In our study, we found MAPK signaling pathway genes, including *mapk3*, *mapk8b*, *mapk14a*, and *raf1* in the model group, were significantly downregulated, and *egfr*, *akt2* were significantly upregulated. *akt1* and *gsk3β* Upregulated but not different. However, GA reversed the regulation effectively. In addition, the expression of *tyr*, *tyrp1a*, *tyrp1b*, *mitf*, *dct*, and *oca2* was significantly upregulated after GA intervention, and there was no difference in *silv*. Therefore, we concluded that GA ameliorated PTU-induced depigmentation by regulating the p38/JNK MAPK signaling pathway.

The B16F10 melanoma cell line has been widely used as an *in vitro* model to regulate melanin. The results showed that GA (1 and 2 μM) treatment 24 did not affect cell viability. In addition, after intervention with GA, the melanin synthesis in B16F10 cells was found to increase. In contrast, the melanin content in the model group was significantly reduced compared to the blank control group. These results were consistent with the findings observed in the zebrafish study conducted *in vivo*.

According to literature reports, MITF is involved in several signaling pathways in melanocytes, such as MAPK, cAMP/PKA, PI3K/AKT, SCF/c-Kit, etc., and is a key factor in regulating melanin biosynthesis (Zhou et al., 2021). The expression of MITF is associated with the activation of upstream MAPK signaling pathways, including p38, JNK, and ERK1/2, which are important pathways involved in melanin biosynthesis (Hu et al., 2019). Studies have shown that the activation of p38 and JNK can upregulate the expression of MITF and then induce melanocyte differentiation and melanin synthesis (Sun et al., 2020; Han and Hyun, 2023). However, the signaling pathway of ERK is controversial in terms of the expression of MITF. The activation of ERK upregulates the expression of MITF (Mamat et al., 2018), and the activation of ERK promotes the degradation of MITF (Son et al., 2011). Therefore, we detected the activation of the MAPK pathway in cells after the intervention of GA. Western blot results showed that GA (1 and 2 μM) increased the phosphorylation levels of p38 and JNK but had no effect on the phosphorylation of ERK. However, the cascade reaction of P38 and JNK MAPK can activate the expression of MITF, which is also a necessary factor to regulate downstream TYR, TRP1, and TRP2. Therefore, phosphorylation of p38 and JNK can directly regulate the expression of TYR and participate in melanin synthesis. Our data showed that activation of phosphorylation of p38 and JNK induced the expression of MITF, TYR, TRP1, anchend TRP2. These results indicated that GA promoted melanin production by activating the P38 MAPK/JNK signaling pathway and then upregulated the expression of MITF, TYR, TRP1, and TRP2.

## 5 Conclusion

In conclusion, our results suggest that GA can downregulate oxidative stress and attenuate melanocyte damage by activating the p38-JNK MAPK signaling pathway and the expression levels of tyrosine-related genes and proteins, thereby promoting melanin synthesis. In additional, based on the results of transcriptomics analysis, we have also deeply investigated the target genes of GA that regulate melanogenesis. It provides experimental basis and theoretical support for the further development of GA as a therapeutic drug for pigment loss.

## Data Availability

The datasets presented in this study can be found in online repositories. The names of the repository/repositories and accession number(s) can be found in the article/Supplementary Material.
