# Supplementary figures and images for: Galangin ameliorates PTU-induced vitiligo in zebrafish and B16F10 cells by increasing melanogenesis through activation of the p38/JNK MAPK pathway
Source: Front Pharmacol. 2025 Mar 10;16:1521097. doi: 10.3389/fphar.2025.1521097 (PMC11931063; doi:10.3389/fphar.2025.1521097)

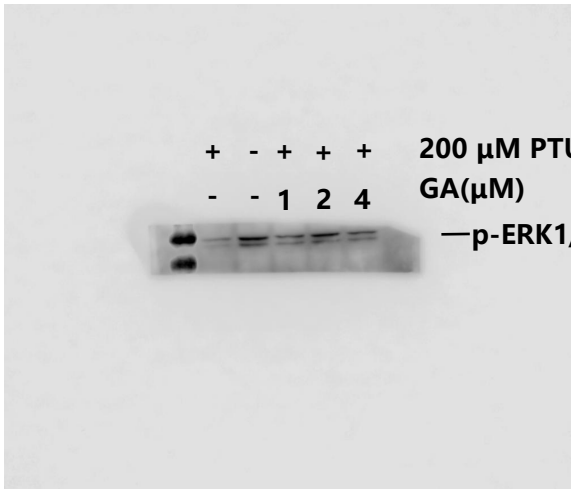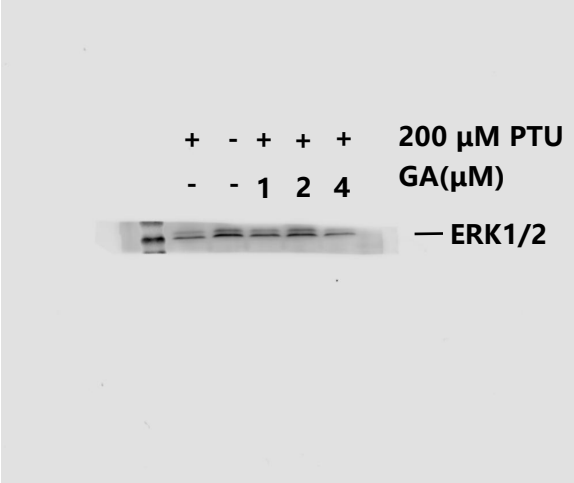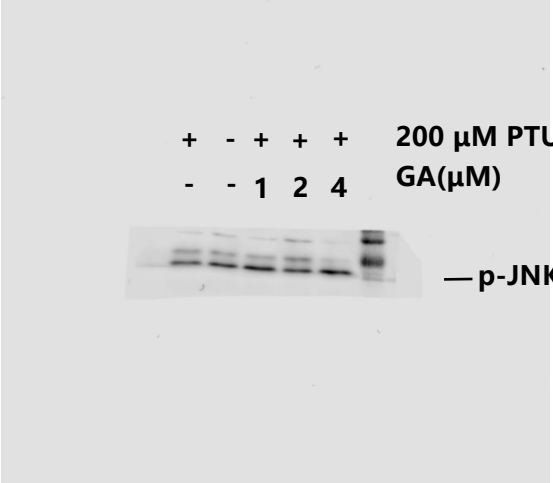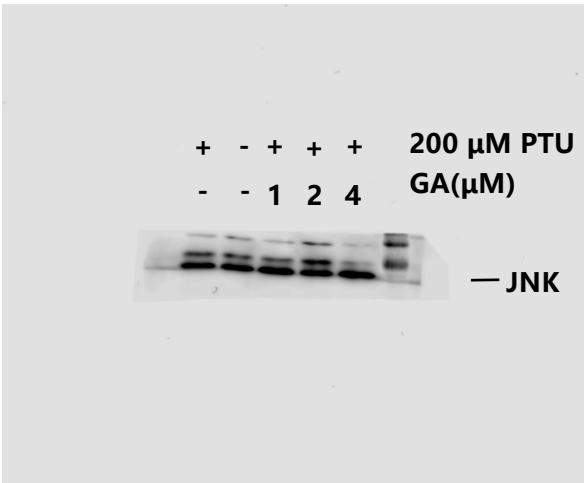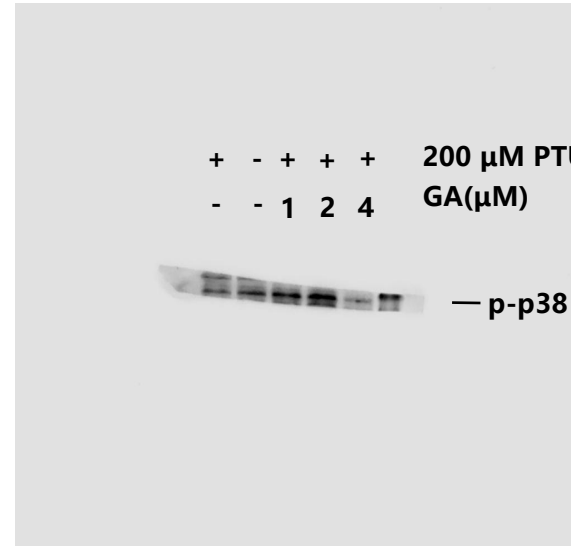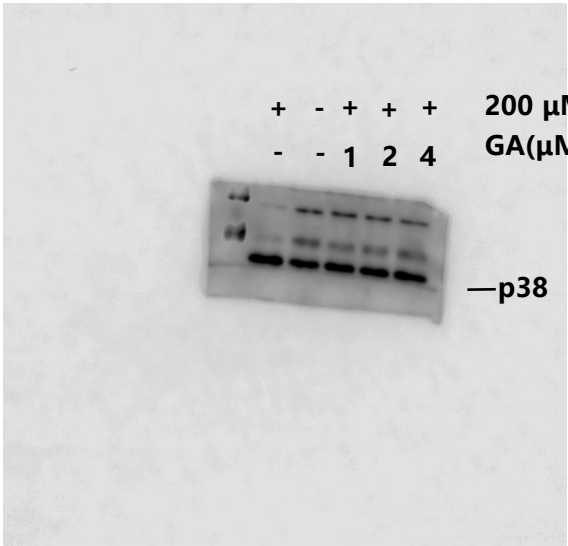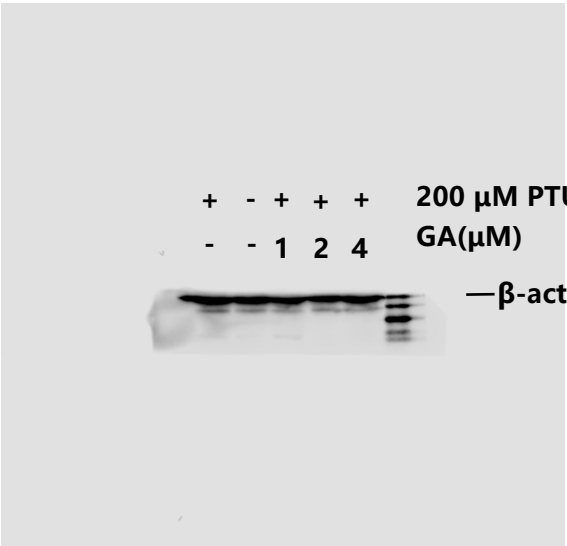

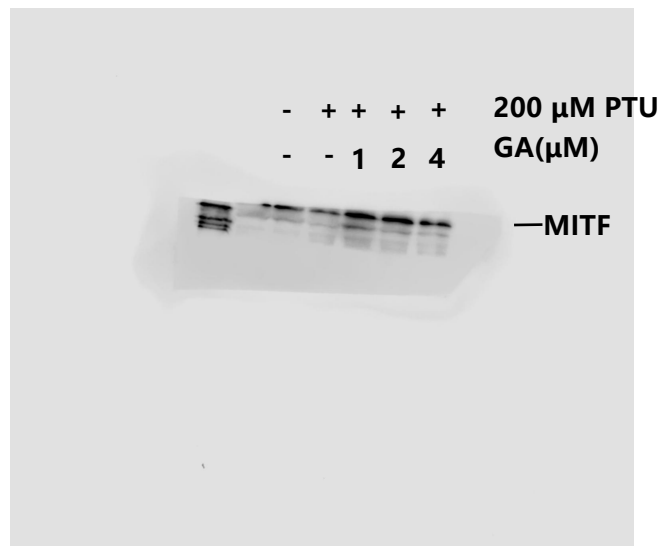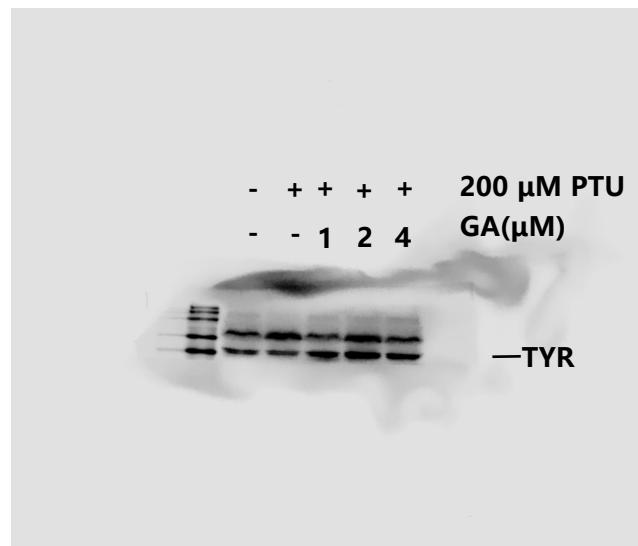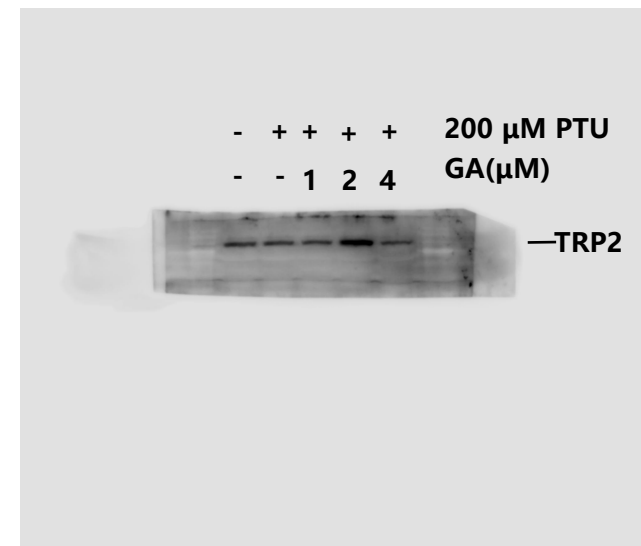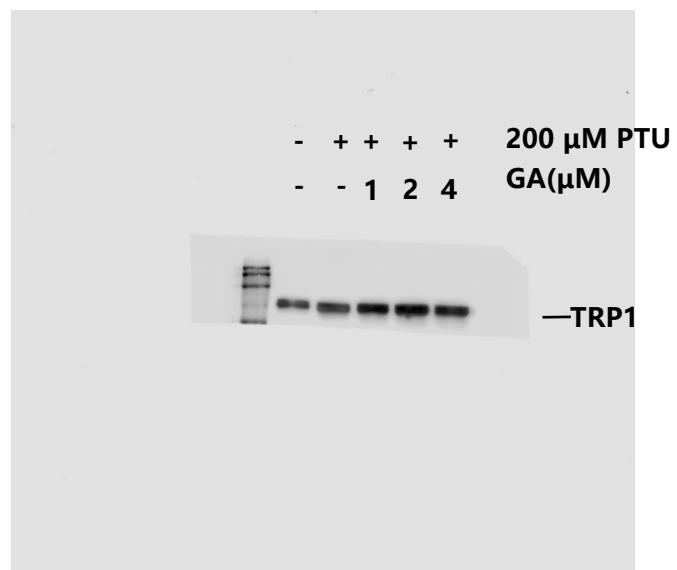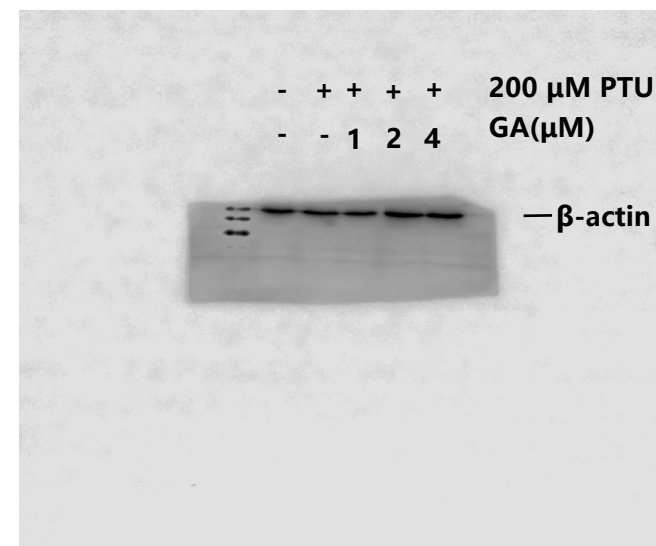

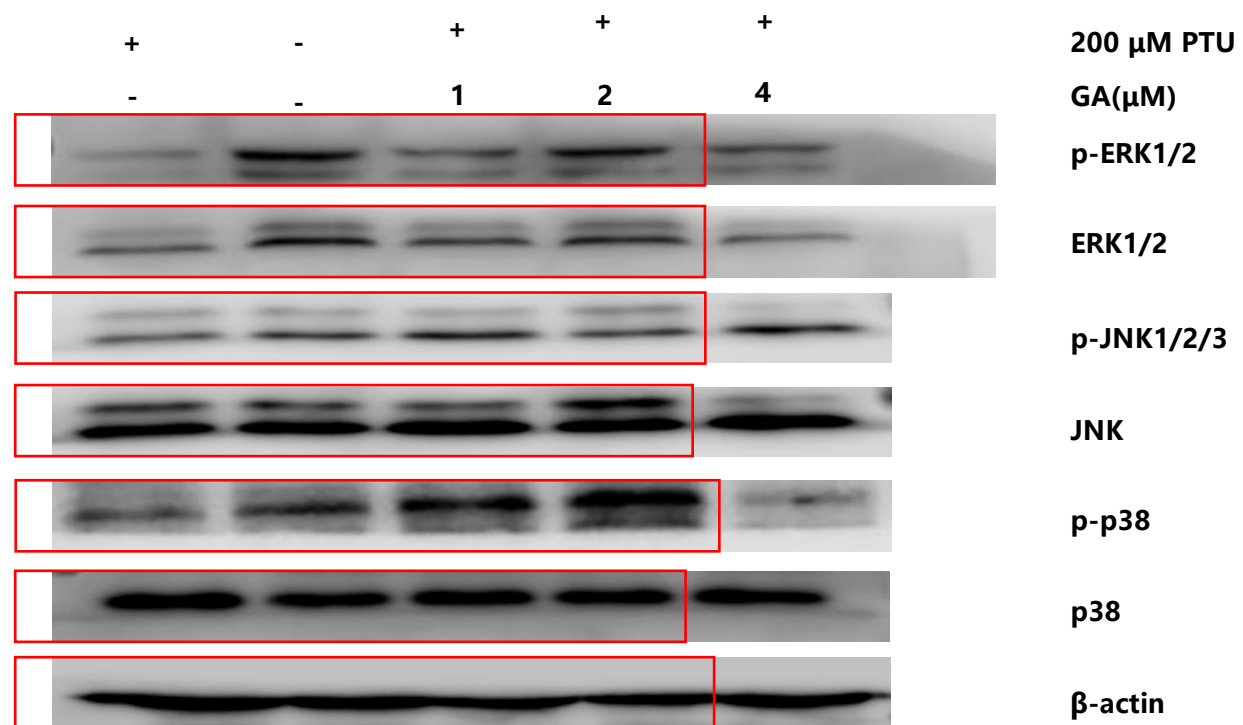

200 μM PTU

GA(μM)

p-ERK1/2

ERK1/2

p-JNK1/2/3

JNK

p-p38

p38

β-actin

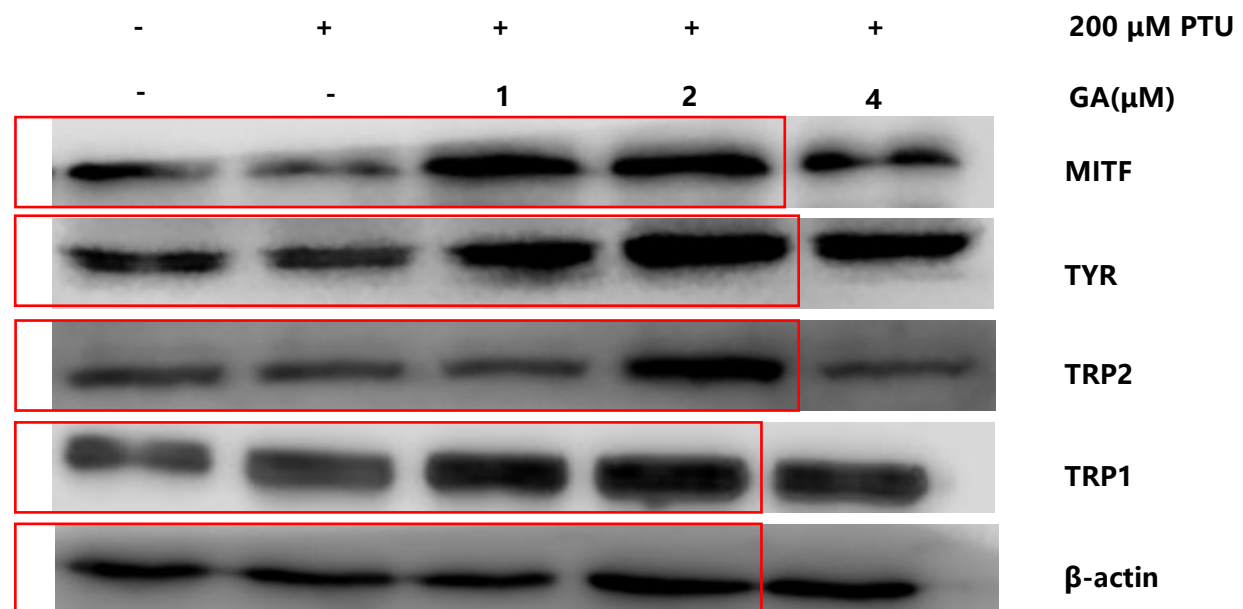

200 μM PTU

GA(μM)

MITF

TYR

TRP2

TRP1

β-actin

Supplement: Supplementary file 1 [file DataSheet1.pdf]
